# Rheumatic Fever Revealed by Pulmonary Involvement: A Case Report

**DOI:** 10.7759/cureus.37334

**Published:** 2023-04-09

**Authors:** Ahmed Mougui, Imane El Bouchti

**Affiliations:** 1 Department of Rheumatology, Ar-Razi Hospital, Faculty of Medicine and Pharmacy of Marrakech, Mohammed VI University Hospital, Marrakech, MAR

**Keywords:** diagnosis, corticosteroid, rheumatic fever, arthritis, pneumonia

## Abstract

Rheumatic fever (RF) is a significant public health problem in underdeveloped countries, and its diagnosis is based on modified Jones criteria. However, there are rare manifestations not included in these criteria that can complicate this condition. We present a case report of a 21-year-old Moroccan female with RF revealed by pulmonary involvement. The patient had no known rheumatic fever. She presented with a two-week history of joint pain, severe chest pain, and shortness of breath. On clinical examination, she was febrile with a palpable left knee joint effusion. Laboratory tests indicated elevated levels of inflammation markers and moderate hepatic cytolysis. The thoracic CT scan revealed extensive bilateral alveolar-interstitial parenchymal involvement. The left knee joint puncture showed an inflammatory fluid without germs or microcrystals. Antibiotic therapy with ceftriaxone and gentamycin was ineffective. Echocardiography revealed rheumatic poly valvulopathy with mitral valve narrowing and moderate to severe mitral insufficiency. Streptolysin O antibody levels were high. The diagnosis of RF complicated by rheumatic pneumonia was made. Treatment with amoxicillin and prednisone led to favorable outcomes.

## Introduction

Rheumatic fever (RF) is an inflammatory disease with an infectious origin, triggered by an autoimmune response to infection with group A *Streptococcus* [1]. The prevalence of RF has decreased considerably since the introduction of recent antibiotics [2]. However, in poorer countries, it remains a frequent cause of morbidity and mortality [1]. Clinical manifestations include joint, cardiac, skin, and sometimes neurological involvement [1]. Pulmonary involvement is a rare and poorly understood complication [3]. This report is of a case of RF revealed by pulmonary involvement in a 21-year-old Moroccan female.

## Case presentation

**Figure 1:**
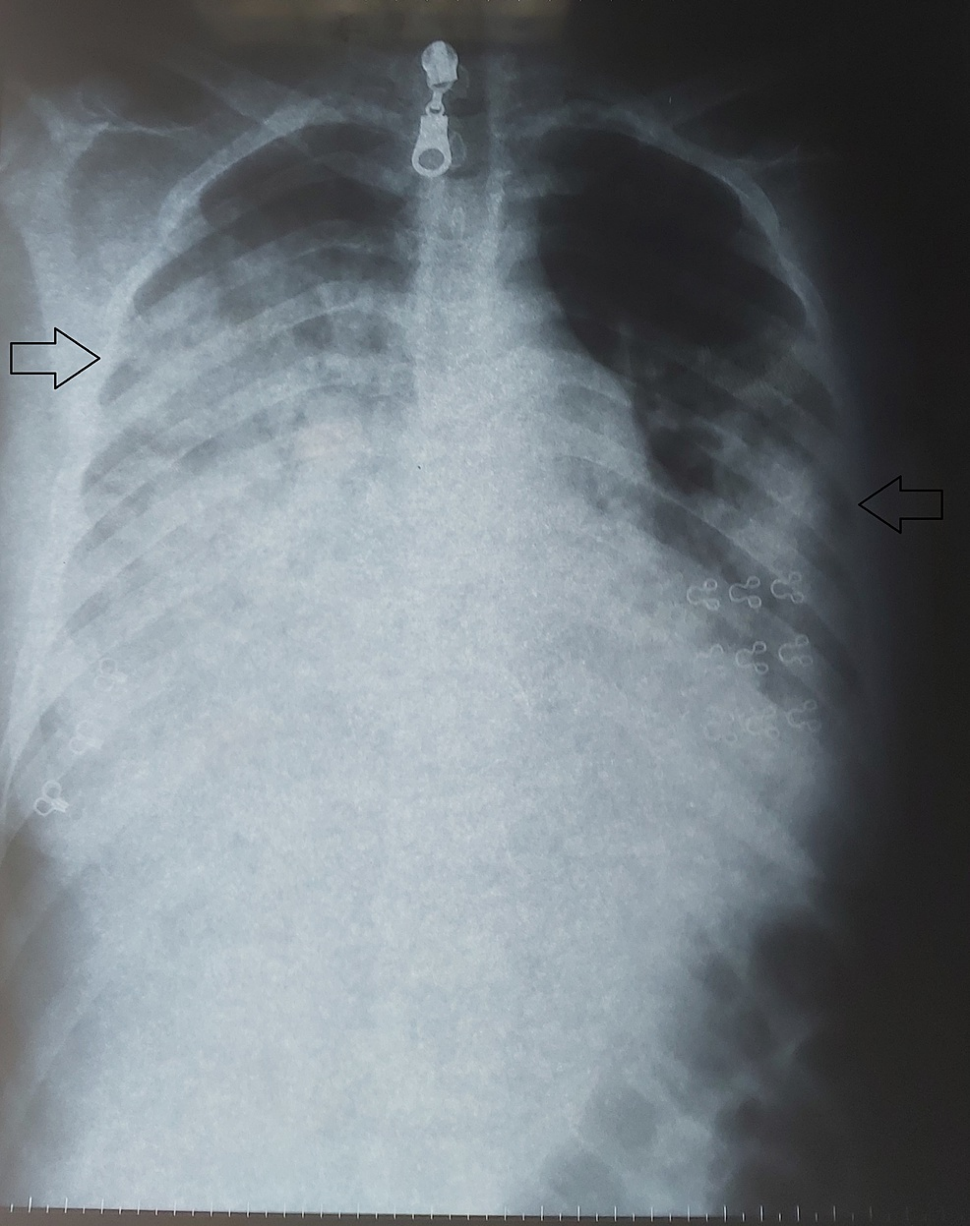
Posteroanterior chest radiograph demonstrates extensive alveolar and interstitial opacities

**Figure 2:**
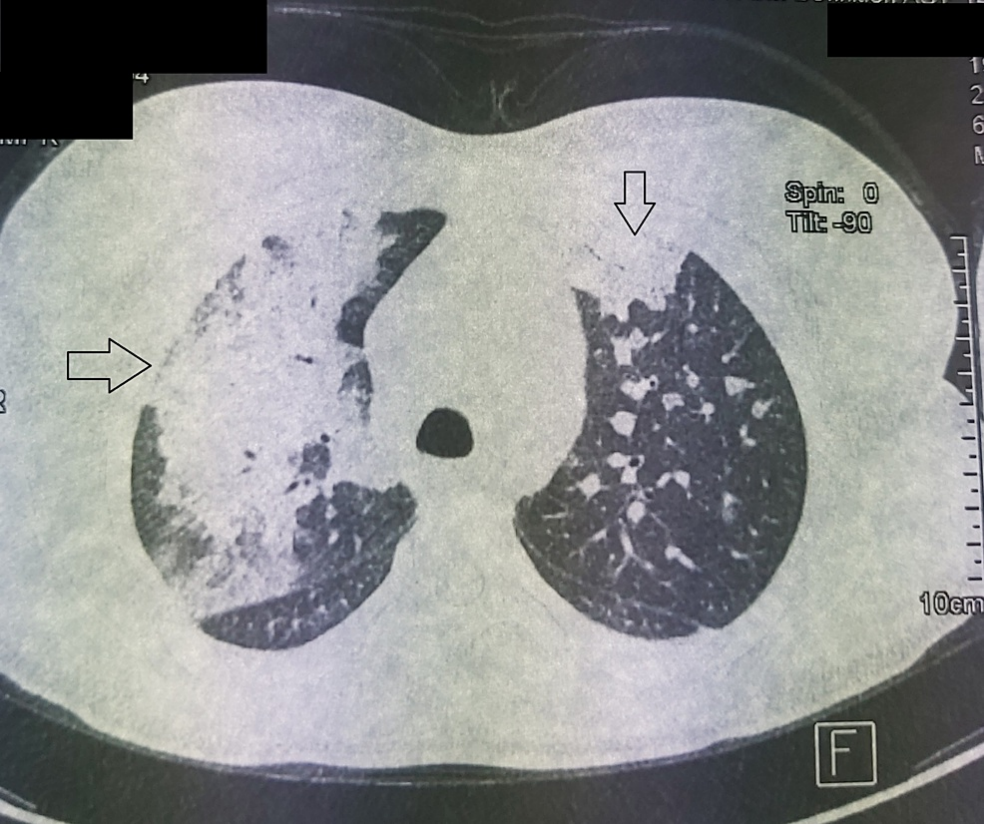
Chest CT axial section, showing bilateral alveolar-interstitial involvement

A 21-year-old Moroccan female presented with a medical history of frequently experiencing strep throat. The last episode, 14 days ago, was untreated, and since then she had been experiencing increasingly severe joint pain in her knees and ankles. Two days ago, she also started experiencing severe chest pain and shortness of breath. During the clinical examination, her body temperature was found to be high at 39°C, her heart rate was fast at 100 beats per minute, and her breathing rate was rapid at 30 cycles per minute. Her oxygen saturation was measured to be 90% while breathing room air. An examination of her joints showed swelling in her left knee. Chest radiograph (Figure 1) and chest computed tomography (Figure 2) showed extensive alveolar and interstitial opacities.

Blood tests showed that she had normochromic anemia with hemoglobin at 11 g/dl, a high white blood cell count with an abundance of neutrophils, high levels of C-reactive protein, and elevated levels of alanine aminotransferase and Aspartate aminotransferase. Tests for coronavirus disease 2019 (COVID-19) were negative. All laboratory values are shown in Table 1. 

**Table 1:** Summary of laboratory results MCV: Mean corpuscular volume; CRP: C-reactive protein; ESR: Erythrocyte sedimentation rate; ALT: Alanine aminotransferase; AST: Aspartate aminotransferase; LDH: Lactate dehydrogenase; ALP: Alkaline phosphatase; ANA: Anti-nuclear antibody;  Anti-CCP: Anti-cyclic citrullinated peptide antibodies; RF: Rheumatoid factor; ANCA:  Anti-neutrophil cytoplasmic antibody;  HIV: Human immunodeficiency virus; HSV: Herpes simplex virus; CMV: Cytomegalovirus

Laboratory test	Result	Normal values
Hemoglobin (g/dl)	11	13-16
MCV	83	85-95
Leukocytes(/mm^3^)	15600	4000-10000
Neutrophils (/mm^3^)	12000	2000-7500
Lymphocytes (/mm^3^)	2000	1500-4000
Platelets (/mm3)	300000	150000-450000
CRP (mg/l)	103	0-5
ESR (mm/hour)	80	0-10
Ferritin (ng/ml)	300	28-365
Creatinine (mg/l)	5	7-12
ALT (U/L)	150	10-41
AST (U/L)	140	10-50
LDH (U/L)	90	0-250
ALP (U/L)	80	35-104
ANA	negative	<1/160
Anti-CCP	8	<25
RF (UI/ML)	5	<16
ANCA	negative	<1/20
HLA B27	negative	-
Syphilis serology	negative	-
HIV serology	negative	-
CMV serology	negative	-
Hepatitis B serology	negative	-
Hepatitis C serology	negative	-
streptolysin O antibody levels (IU/L)	527	<150
Procalcitonin (ng/ml)	0,03	<0,5

**Figure 3:**
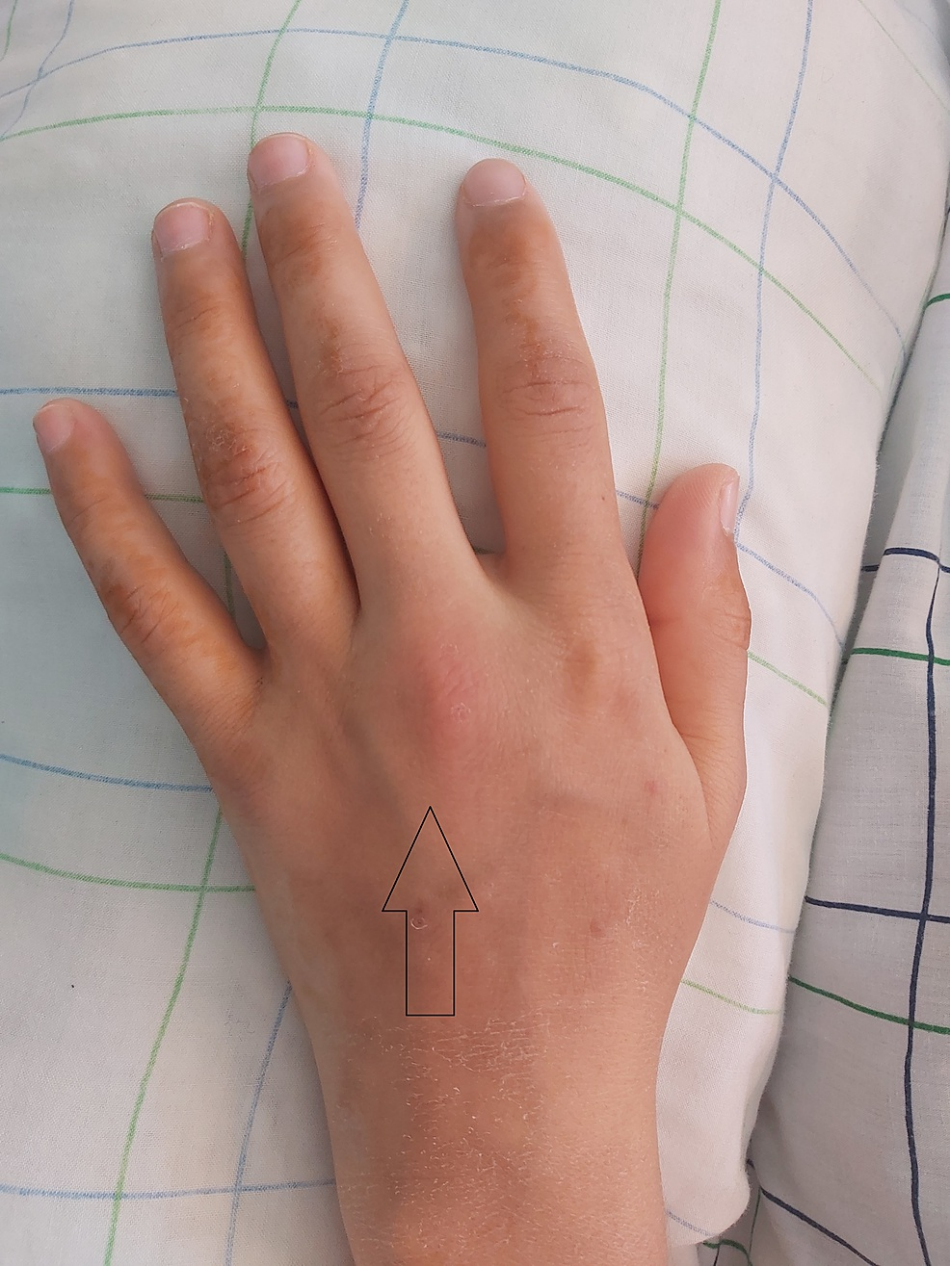
Joint effusion of the third left metacarpophalangeal joint

The patient was treated with two antibiotics, ceftriaxone and gentamycin, to address a suspected case of bacterial pneumonia complicated by septic arthritis. However, after three days, her respiratory and joint symptoms worsened, and the inflammation persisted. Further examination showed joint effusion in her third left metacarpophalangeal joint (Figure 3), knees, and ankles.

**Figure 4:**
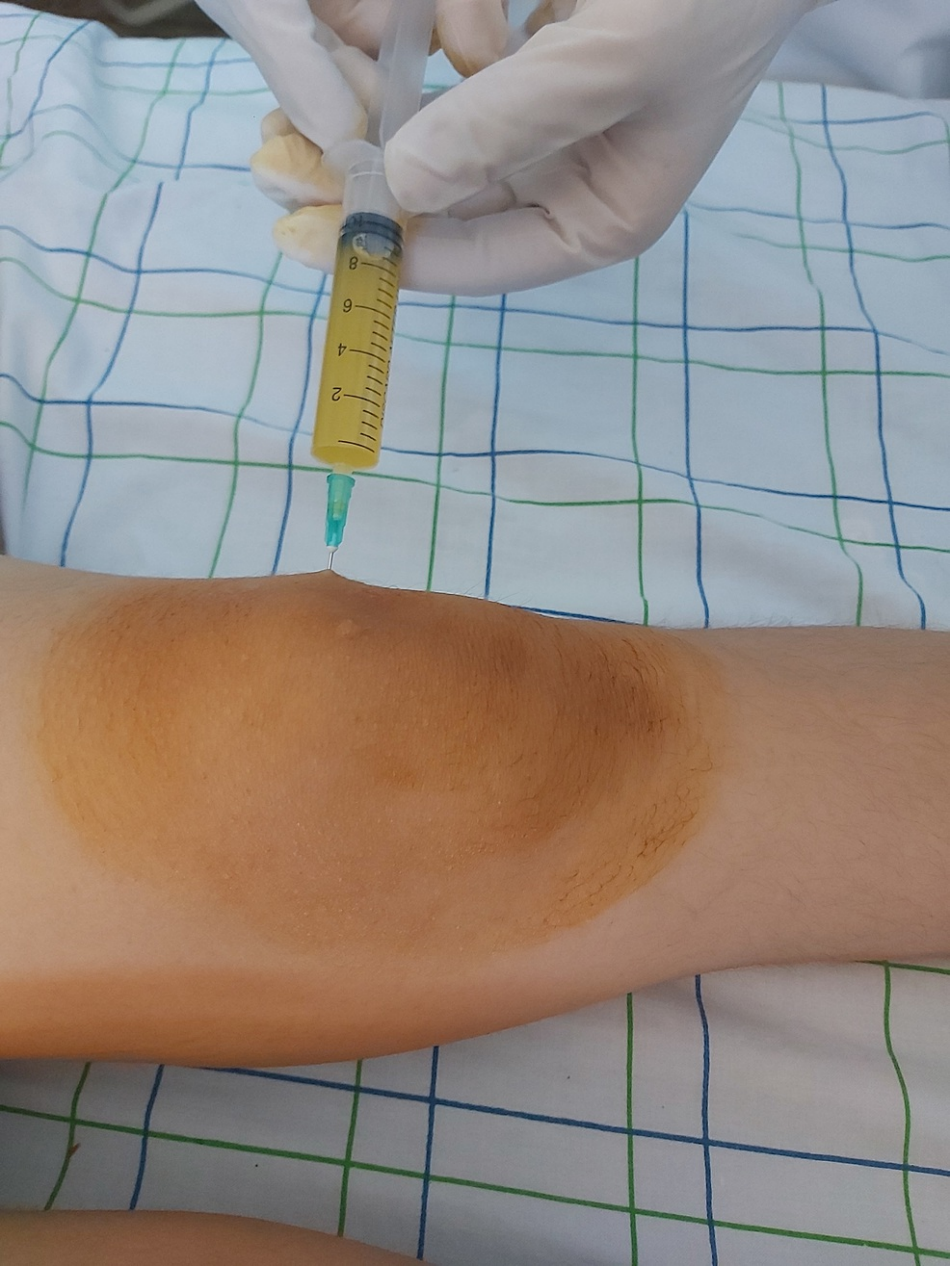
Citrine yellow fluid at the articular puncture

Arthrocentesis of the left knee yielded an inflammatory fluid (Figure 4) with leukocytes at 2000 elements/mm^3^, without germs or microcrystals. Blood cultures were negative.

Radiological examinations did not reveal any signs of rheumatic pathology, but echocardiography showed rheumatic poly valvulopathy with mitral valve narrowing and moderate to severe mitral insufficiency. Streptolysin O antibody levels were high at 527 IU/L. The patient was diagnosed with RF complicated by rheumatic pneumonia and was treated with amoxicillin 100 mg/kg/d and prednisone 60 mg/d, which led to complete regression of her symptoms, including her joint pain, respiratory symptoms, and inflammation (Table 2).

**Table 2:** Summary of follow-up laboratory testing after treatment MCV: Mean corpuscular volume; CRP: C-reactive protein; ESR: Erythrocyte sedimentation rate; ALT: Alanine aminotransferase; AST: Aspartate aminotransferase; LDH: Lactate dehydrogenase; ALP: Alkaline phosphatase

Laboratory test	Result	Normal values
Hemoglobin (g/dl)	14	13-16
MCV	86	85-95
Leukocytes(/mm^3^)	8000	4000-10000
Neutrophils (/mm^3^)	4000	2000-7500
Lymphocytes (/mm^3^)	2000	1500-4000
Platelets (/mm3)	300000	150000-450000
CRP (mg/l)	17	0-5
ESR (mm/hour)	20	0-10
Ferritin (ng/ml)	103	28-365
Creatinine (mg/l)	5	7-12
ALT (U/L)	30	10-41
AST (U/L)	40	10-50
LDH (U/L)	90	0-250
ALP (U/L)	80	35-104

**Figure 5:**
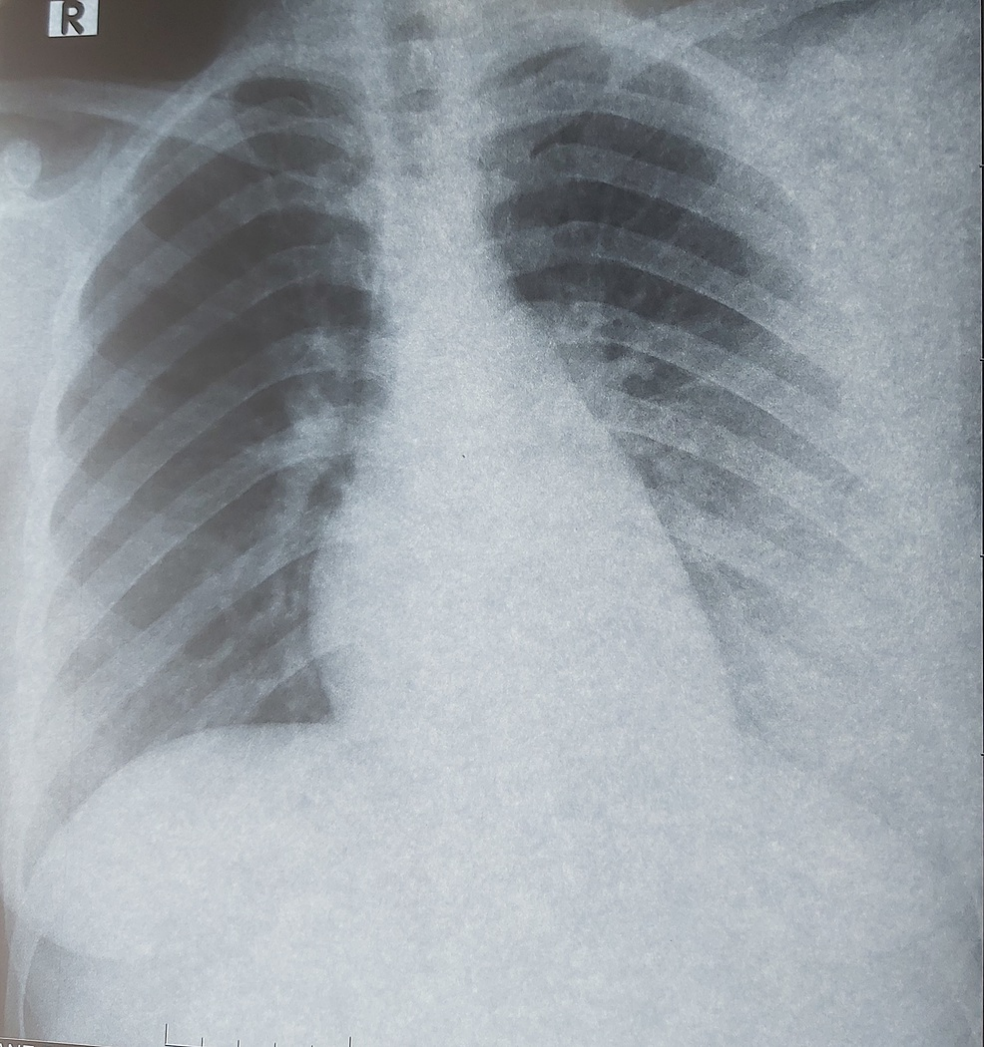
Control chest X-ray, showing improvement of lung involvement under corticosteroid therapy

The chest X-ray after 15 days of treatment showed improvement in pulmonary involvement (Figure 5).

The degression of corticosteroid therapy was initiated in the third week and secondary antibiotic prophylaxis based on benzathine penicillin 1.2 MUI every 21 days was started. Follow-up examinations after six months showed good clinical and biological evolution.

## Discussion

RF, a common autoimmune disease in developing countries, is often secondary to infection with group A beta-hemolytic streptococcus. In 1944, Jones described the five classic manifestations of RF, including arthritis, carditis, erythema marginata, subcutaneous nodules, and chorea, which practitioners have retained with minor modifications [4]. However, there may be other non-specific manifestations accompanying or revealing RF [5]. Pulmonary involvement during RF is a rare and poorly understood complication, which may be primary or secondary to cardiac involvement, uremia, or an intercurrent infection [6]. Rheumatic pneumonia affects about 2% of patients, most often with carditis, and remains a diagnosis of elimination [7]. The clinical picture is that of acute pneumonia, with dyspnea, non-productive cough, fever, and varying degrees of chest pain [3]. Several radiographic aspects have been described, including nodular involvement, diffuse infiltrates, pleural effusions, and condensation areas [6]. No pathological aspect is specific, and several lesions have been reported, including alveolar hemorrhages, septal necrosis, and Masson's bodies [8]. Corticosteroid therapy appears to be the most effective treatment for rheumatic pneumonia [6,9]. However, deaths can occur despite steroid therapy [10]. Our patient fulfills the modified Jones criteria of RF [11], and the echocardiographic appearance suggests unrecognized RF. Moderate hepatic cytolysis has been described in RF [5,12]. The diagnosis of rheumatic pneumonia secondary to RF was retained in our patient, given the clinical, biological, and radiological presentation compatible with cases described in the literature, the negative assessment of other etiologies, and the spectacular evolution under corticosteroid therapy [13].

## Conclusions

Rheumatic pneumonia presents a diagnostic challenge for practitioners who must always consider this diagnosis when faced with pneumonia that does not respond to antibiotics, especially for patients living in regions endemic to RF. Primary prevention, which is based on improving socio-economic conditions and access to care, is the most effective strategy for preventing RF and its complications, which can lead to significant morbidity and early mortality.
